# Pan-cancer structurome reveals overrepresentation of beta sandwiches and underrepresentation of alpha helical domains

**DOI:** 10.1038/s41598-023-39273-5

**Published:** 2023-07-25

**Authors:** Kirill E. Medvedev, R. Dustin Schaeffer, Kenneth S. Chen, Nick V. Grishin

**Affiliations:** 1 Department of Biophysics, University of Texas Southwestern Medical Center, Dallas, TX 75390 USA; 2 Department of Pediatrics, University of Texas Southwestern Medical Center, Dallas, TX 75390 USA; 3 Children’s Medical Center Research Institute, University of Texas Southwestern Medical Center, Dallas, TX 75390 USA; 4 Department of Biochemistry, University of Texas Southwestern Medical Center, Dallas, TX 75390 USA

**Keywords:** Bioinformatics, Computational biology and bioinformatics, Structural biology

## Abstract

The recent progress in the prediction of protein structures marked a historical milestone. AlphaFold predicted 200 million protein models with an accuracy comparable to experimental methods. Protein structures are widely used to understand evolution and to identify potential drug targets for the treatment of various diseases, including cancer. Thus, these recently predicted structures might convey previously unavailable information about cancer biology. Evolutionary classification of protein domains is challenging and different approaches exist. Recently our team presented a classification of domains from human protein models released by AlphaFold. Here we evaluated the pan-cancer structurome, domains from over and under expressed proteins in 21 cancer types, using the broadest levels of the ECOD classification: the architecture (A-groups) and possible homology (X-groups) levels. Our analysis reveals that AlphaFold has greatly increased the three-dimensional structural landscape for proteins that are differentially expressed in these 21 cancer types. We show that beta sandwich domains are significantly overrepresented and alpha helical domains are significantly underrepresented in the majority of cancer types. Our data suggest that the prevalence of the beta sandwiches is due to the high levels of immunoglobulins and immunoglobulin-like domains that arise during tumor development-related inflammation. On the other hand, proteins with exclusively alpha domains are important elements of homeostasis, apoptosis and transmembrane transport. Therefore cancer cells tend to reduce representation of these proteins to promote successful oncogeneses.

## Introduction

How to discover proteins’ biological functions has long been one of the key questions of both experimental and computational research. The 3D structures of proteins, which are determined by their amino acid sequence, determines protein function. Protein domains serve as structural, functional, and evolutionary units; classifying and understanding their evolutionary relationships can be challenging. Our Evolutionary Classification of protein Domains (ECOD) is a hierarchical evolutionary classification, which in comparison to other structure-based domain classifications groups domains foremost by homology, rather than topology^1,2^. This feature helps to identify cases of homology between domains that have different topologies. Another important feature of ECOD is its emphasis on distant homology, resulting in a catalog of evolutionary relationships between classified domains.

Cancer is a complex and heterogeneous disease that requires a comprehensive (pan-cancer) approach. Pan-cancer studies explore the common characteristics and variations across a wide range of tumor types^3^ and have been conducted at multiple levels of molecular organization: genomic^4^, transcriptomic^4^, proteomic^5^, lncRNAs^6^, among others. However, the structural aspect of cancer related proteins has never been studied on a large-scale. AlphaFold (AF)—a recently developed deep learning method by DeepMind, demonstrated the capability to predict protein structure with atomic-level accuracy^7^. Application of AF to proteins without a known experimental structure has significantly increased the proportion of proteins with accurately predicted structures, including those within the human proteome^8^. However, AF models have variable quality, with significant differences in reliability across different regions of the protein chain. Thus, it is crucial to use these models with caution and to have a comprehensive understanding of their limitations^9^. Domains from AF models for the whole human proteome were classified in a special version of ECOD^10^. Here we studied and evaluated the pan-cancer structurome—the structural space of proteins over and underexpressed in 21 cancer types from The Cancer Genome Atlas (TCGA) using domains from the ECOD classification. In both sets we examined overrepresented proteins whose abundance in cancer sets is significantly higher than in the human proteome in general, and thus are highly relevant for oncogenesis; and underrepresented proteins that are less common in cancer than in the whole proteome. We showed that AF models significantly expand the 3D structural space of proteins differentially expressed in 21 cancer types. Analysis of top-level ECOD architecture groups (A-groups) revealed significant overrepresentation of beta sandwich domains and underrepresentation of alpha helical domains for the majority of cancer types. We suggest that overrepresentation of beta sandwiches is related to the abundance of immunoglobulins and immunoglobulin-like domains due to inflammation that accompanies tumor development. Conversely, proteins with exclusively alpha domains play critical roles in maintaining cellular homeostasis, regulating apoptosis, and facilitating transmembrane transport. Cancer cells tend to decrease the representation of these proteins. This decrease is a strategy employed by cancer cells to promote successful oncogenesis, potentially by disrupting normal cellular processes associated with homeostasis, apoptosis, and transmembrane transport.

Domain classifications such as ECOD, SCOP, and CATH contain broad levels in their classification that describe amounts and arrangements of secondary structure in their constituent domains^11–13^. The relationship between the evolution of protein topology and consequent possible functions of those topologies remains murky and an area of active investigation^14^. In ECOD we maintain our 21 “architecture” (A-group) levels as a method of broadly classifying the secondary structure content of domains, their general arrangement, and their possible functions. The architecture level is maintained by expert curation and is not the subject of automated approaches. ECOD architectures are inherited (in part) from SCOP classes^15,16^. For example, we add additional architectures to distinguish between alpha arrays and bundles, as well as to separate those domains that participate in obligate multimer activity. Additionally, we maintain “special” architectures to hold those regions of protein that are difficult to classify by homology (e.g., coiled coils) or that are not the product of evolution (e.g. de novo synthetic domains or fragments arising from experimental protein constructs). Here we show how function is distributed among ECOD architectures in the case of protein-coding genes over- and underrepresented in 21 human cancer types.

## Results and discussion

### AlphaFold models significantly expanded structural space of over and underexpressed protein-coding genes in 21 cancer types

Our non-redundant sets of over and underexpressed protein-coding genes for all cancer types include 5341 and 7320 genes, respectively.

**Figure 1:**
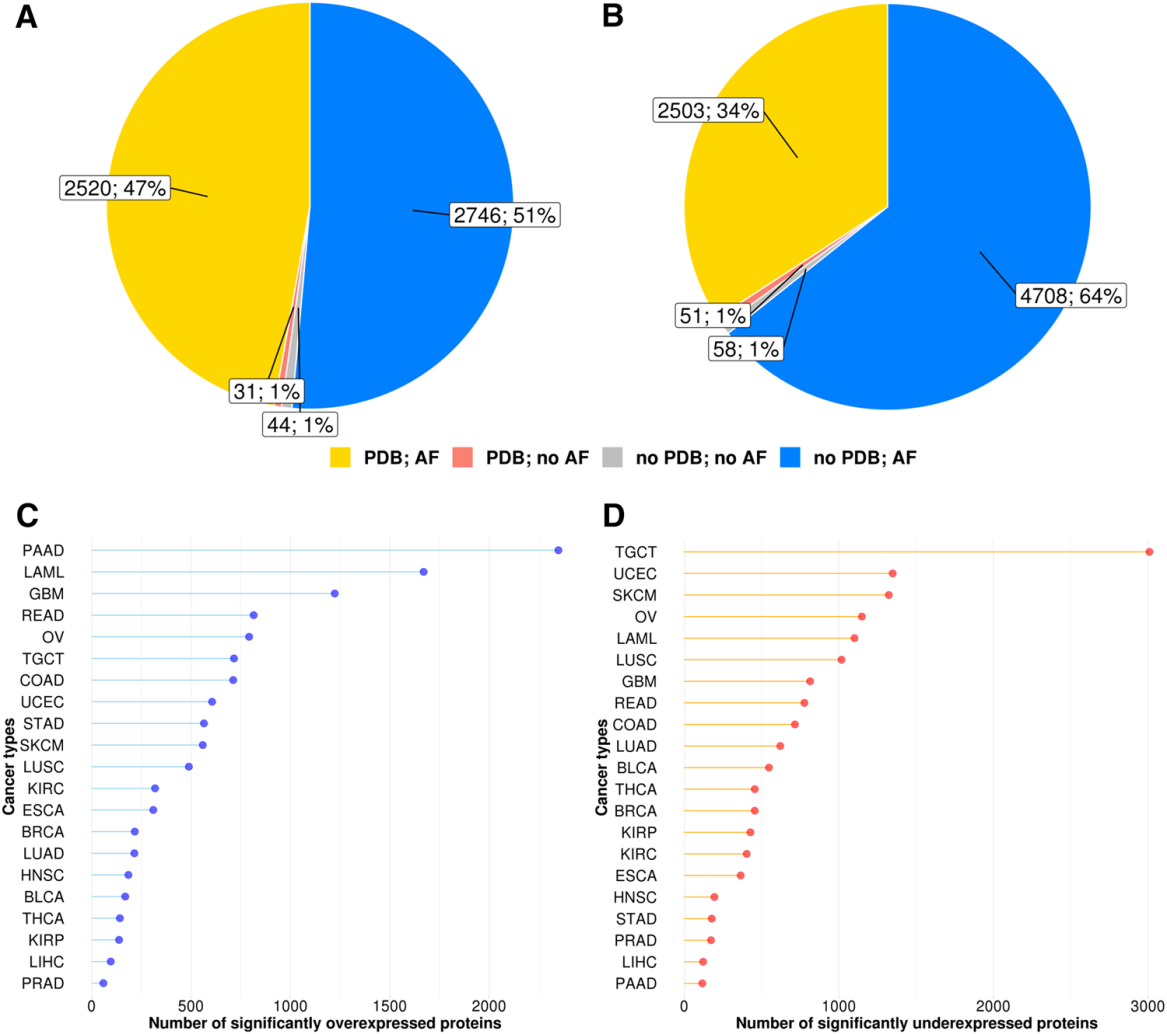
Over and underexpressed protein-coding genes statistics. (**A**) Availability of known 3D protein structures (PDB) and AlphaFold models (AF) for overexpressed protein-coding genes in all cancer types. (**B**) Availability of known 3D protein-coding genes structures (PDB) and AlphaFold models (AF) for underexpressed protein-coding genes in all cancer types. Number of significantly over (**C**) and underexpressed protein-coding genes (**D**) in each cancer type. BLCA, bladder urothelial carcinoma; BRCA, breast invasive carcinoma; COAD, colon adenocarcinoma; ESCA, esophageal carcinoma; GBM, glioblastoma multiforme; HNSC, head and neck squamous cell carcinoma; KIRC, kidney renal clear cell carcinoma; KIRP, kidney renal papillary cell carcinoma; LAML, acute myeloid leukemia; LIHC, liver hepatocellular carcinoma; LUAD, lung adenocarcinoma; LUSC, lung squamous cell carcinoma; OV, ovarian serous cystadenocarcinoma; PAAD, pancreatic adenocarcinoma; PRAD, prostate adenocarcinoma; READ, rectum adenocarcinoma; SKCM, skin cutaneous melanoma; STAD, stomach adenocarcinoma; TGCT, testicular germ cell tumors; THCA, thyroid carcinoma; UCEC, uterine corpus endometrial carcinoma.

Figure 1A, B illustrates the availability of known 3D structures (PDB) and AlphaFold models (AF). For the overexpressed set the fraction of proteins with experimental structures (shown in yellow) and with predicted structures only (shown in blue) are nearly equal (47% and 51% correspondently), whereas for the underexpressed set the fraction of the predicted structures is much higher (64%). Comparing our over and underexpressed protein-coding genes using the GEPIA2 database revealed a significant variation in the number of protein-coding genes whose expression was altered in different cancer types (Fig. 1C, D). This variation might be the result of multiple factors. First, sets of proteins were obtained generated using bulk RNA-sequencing data that includes different cell types, and the fraction of different cell types varies between samples. Second, high heterogeneity between patients were observed for most of the cancer types, which may lead to variation in differentially expressed genes (DEGs) for different sets of samples. Third, different cancers have different rates of cellular differentiation, which means different rate of similarity to the cells of origin (normal cells). DEGs are identified by comparison to normal cells which may also include many cell types and contribute to the variations in DEGs. Finally, the organization of the particular cancer type studies inside TCGA database may contribute to the bias. For example, TCGA-GMB (glioblastoma) includes 599 cancer cases solely diagnosed as “glioblastoma”^17^. However, the TCGA-BRCA (breast cancer) study contains 1097 cases, including several subtypes (infiltrating duct carcinoma, lobular carcinoma, etc.) that present distinct molecular characteristics^18^. So, this additional heterogeneity inside TCGA studies might account for the overall higher number of over and underexpressed proteins found for glioblastoma in comparison to breast cancer.

We used the ECOD^1^ classification of experimental structures and the ECOD human classification^10^ of AlphaFold models (ECOD_AF) to retrieve domain information for sets of over and underexpressed protein-coding genes. ECOD includes five levels of domains hierarchy: architecture (A), possible homology (X), homology (H), topology (T), and family (F). ECOD_AF that includes only human protein structures predicted by AlphaFold. AlphaFold models have been classified to the T-group level. Over and underrepresentation of proteins expressed in different cancer types, which domains belong to particular ECOD A-gr/X-gr, were calculated as ratio of observed and expected frequencies (see “Materials and methods”). ECOD_AF includes 47,576 domains, of which only 23% have been included in experimental structures^10^. 6.3% of these classified globular domains lack sequence-based annotation in InterPro database^19^. The reference human proteome (UNP: UP000005640) was used for identification of the total number of human proteins. The reference human proteome includes 20,385 proteins, 84% (17,172) of which have been classified in ECOD and ECOD_AF. Over and underexpressed sets include 88% (4709 out of 5341) and 84% (6134 out of 7320) of proteins classified in ECOD and ECOD_AF respectively. The main reasons for the absence of a particular protein in ECOD and ECOD_AF are a high fraction of disordered regions, the low quality of its experimental structure, and a low predicted local-distance difference test (pLDDT score) in its AlphaFold model. We checked the distribution of pLDDT scores for protein regions classified as domains versus all other regions across all AF models used in this study. This score provides valuable information regarding the local reliability of the predicted protein structure and exhibits a strong correlation with global measures of quality. As a result, it serves as a robust tool for evaluating the quality of structure predictions^9,20^. Our results showed that pLDDT score for domains is significantly higher than for all other protein regions (SI Fig. 1).

**Figure 2:**
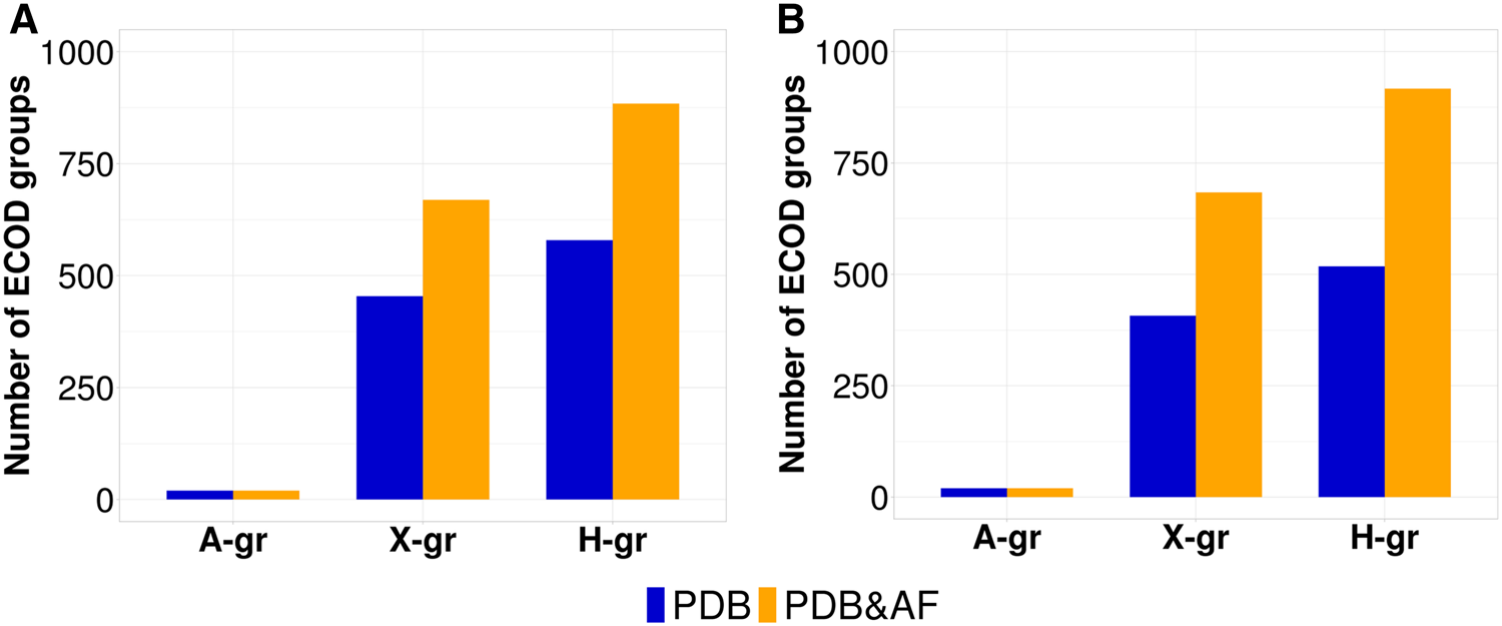
ECOD groups statistics for over and underexpressed protein-coding genes in all cancer types. (**A**) ECOD statistics for overexpressed protein-coding genes. (**B**) ECOD statistics for underexpressed protein-coding genes. Blue bars (PDB) correspond to ECOD domains from experimentally identified structures. Orange bars (PDB&AF) correspond to ECOD and ECOD_AF domains from both experimentally and AlphaFold predicted structures. A-gr, ECOD architecture groups; X-gr, ECOD possible homology groups; H-gr, ECOD homology groups.

Comparison of domains classification statistics of over and underexpressed protein-coding genes before and after AlphaFold structural models release in ECOD and ECOD_AF revealed a more than 1.5-fold increase in the number of X- (possible homology) and H-groups (homology) (Fig. 2). The number of A-groups (architecture), the highest level of ECOD classification, did not change after releasing of the AlphaFold models for human proteome. X- and H-groups are the most important classification levels to consider during identification of distant homology between domains, because similarity at the A-group level does not connotate shared ancestry and may be the result of convergent evolution^2^. Although, there are no newly introduced X- and H-groups in ECOD_AF for over and underexpressed proteins in comparison to ECOD, AlphaFold models significantly expanded 3D structural space for proteins differentially expressed in 21 cancer types. Expansion of the protein structural space inside existing X- and H-groups represent additional opportunities for the search of the potential targets for anticancer therapy.

### ECOD groups reveal overrepresentation of beta sandwiches and underrepresentation of exclusively alpha helical A-groups

**Figure 3:**
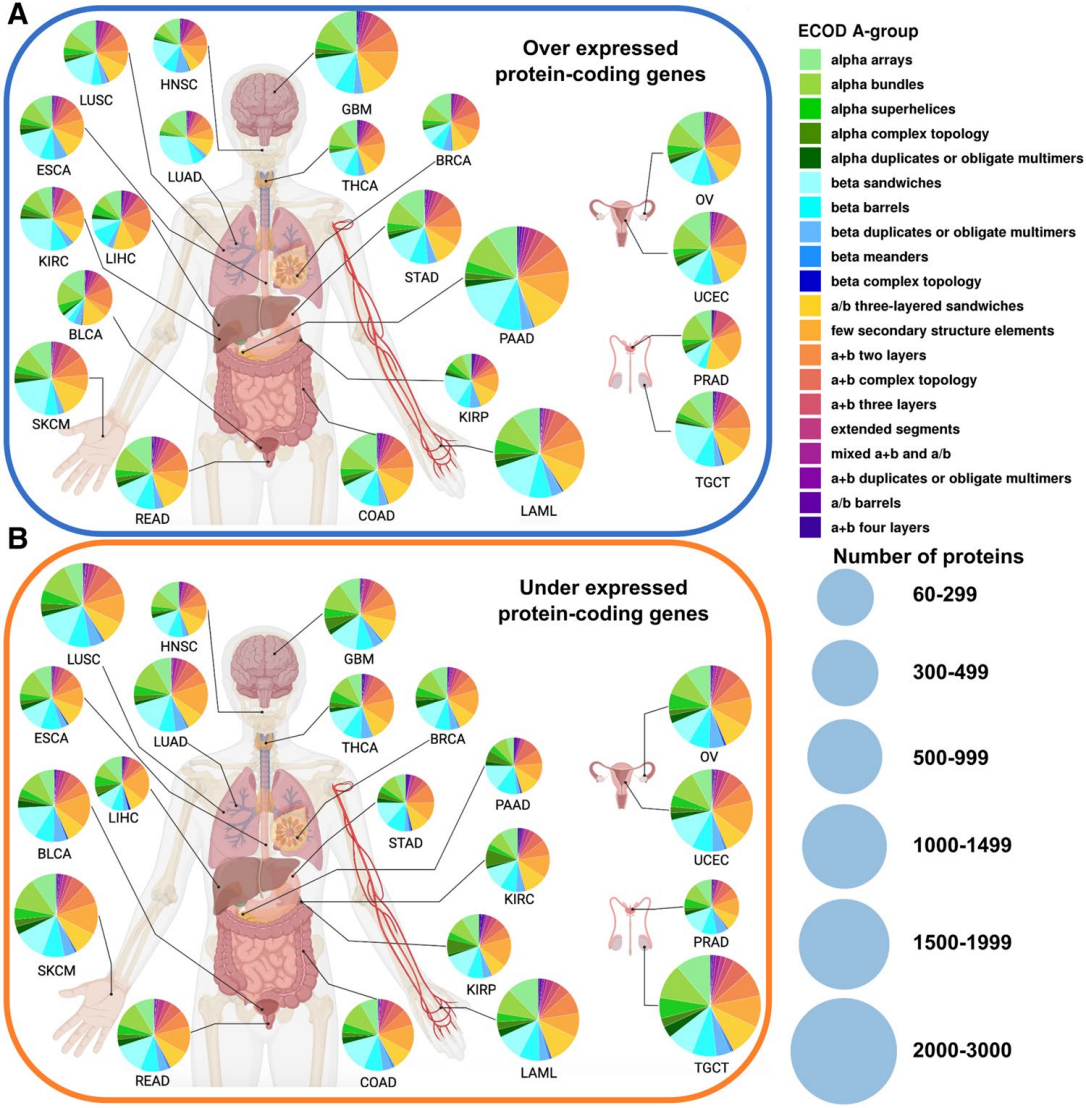
Distribution of cancer-related proteins in ECOD A-groups. (**A**) Protein-coding genes overexpressed in 21 cancer types. (**B**) Protein-coding genes underexpressed in 21 cancer types. The size of each circle correlates to the number of protein-coding genes in a given cancer type. BLCA, bladder urothelial carcinoma; BRCA, breast invasive carcinoma; COAD, colon adenocarcinoma; ESCA, esophageal carcinoma; GBM, glioblastoma multiforme; HNSC, head and neck squamous cell carcinoma; KIRC, kidney renal clear cell carcinoma; KIRP, kidney renal papillary cell carcinoma; LAML, acute myeloid leukemia; LIHC, liver hepatocellular carcinoma; LUAD, lung adenocarcinoma; LUSC, lung squamous cell carcinoma; OV, ovarian serous cystadenocarcinoma; PAAD, pancreatic adenocarcinoma; PRAD, prostate adenocarcinoma; READ, rectum adenocarcinoma; SKCM, skin cutaneous melanoma; STAD, stomach adenocarcinoma; TGCT, testicular germ cell tumors; THCA, thyroid carcinoma; UCEC, uterine corpus endometrial carcinoma. This figure was created with BioRender.com.

ECOD classification levels connotate different probable levels of homology between domains. To evaluate the structurome of the major 21 cancer types, we focused on the two broadest ECOD levels: A-groups (architecture level) and X-groups (possible homology level). The architecture level collects domains with generally similar secondary structure compositions and topologies. The possible homology level brings together domains where some evidence of homology exists but further evidence is needed for certainty of homology. Our protein domain distribution analysis of ECOD A-groups revealed that for overexpressed dataset the “beta sandwiches” A-group has the most prevalent representation in the majority of cancer types (Fig. 3A, SI Fig. 2A).

The exceptions are 8 cancer types: BLCA, BRCA, COAD, LIHC, OV, PRAD, READ, and UCEC. The “alpha arrays” A-group is the most prevalent for 6 out of 8 these cancer types (BLCA—23%, BRCA—20%, COAD—22%, OV—18%, READ—20%, UCEC—20%), whereas the “a + b two layers” and “few secondary structure elements” are the most prevalent for the remaining two (LIHC—20% and PRAD—25% respectively). On the other hand, in the underexpressed set only 10 out of 21 cancer types show beta sandwiches as the most prevalent A-group (Fig. 3B, SI Fig. 2B). In the remaining cancers in the underexpressed set, the most prevalent A-groups are the “few secondary structure elements” (BLCA—25%, KIRP—22%, LIHC—26%, OV—20%, SKCM—20%, THCA—22%, UCEC—23%), “alpha bundles” (ESCA—19%, TGCT—17%), and “a/b three-layered sandwiches” (KIRC—17%, LAML—19%). Overall, for the over and underexpressed sets, the most prevalent five A-groups are “alpha bundles”, “alpha arrays”, “few secondary structure elements”, “a/b three-layered sandwiches” and “beta sandwiches”. The beta sandwiches are the most populated A-group in the human proteome (SI Fig. 3). We believe that the prevalence of beta sandwiches in overexpressed set is the abundance of immunoglobulin and immunoglobulin-like domains that belong to this A-group. Proteins containing these domains are known to be involved in inflammatory processes, which are often significantly upregulated in cancer^21^. This corresponds with the low prevalence of beta sandwiches in the underexpressed set, where less than a half of these cancer types had prevalent beta sandwich representation.

**Figure 4:**
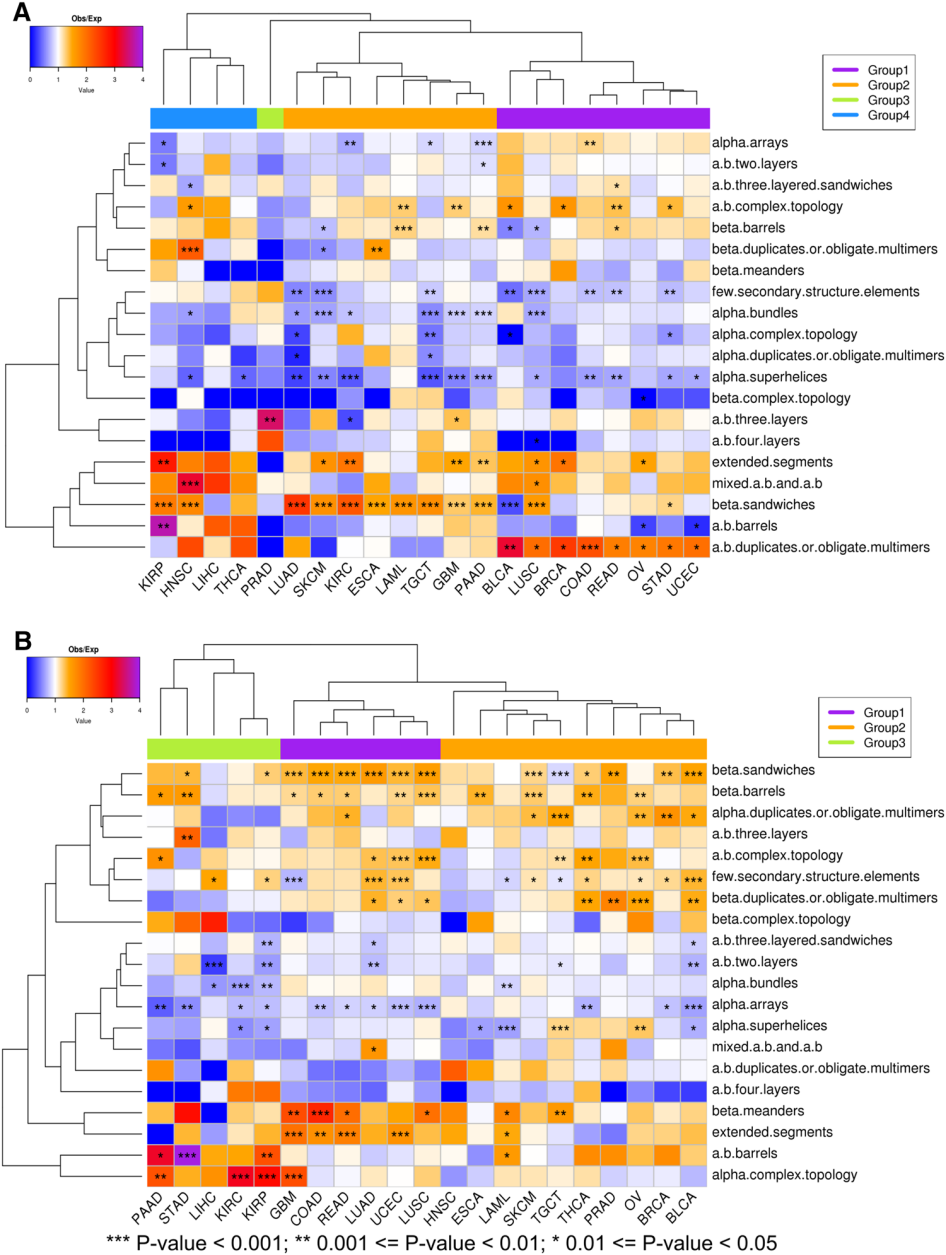
Over and under representation of cancer-related proteins in ECOD A-groups. Heatmap analysis of protein representation in over and underexpressed protein sets. Cells are colored by the ratio of observed to expected frequencies, and ordered on both axes by independent hierarchal clustering. (**A**) Protein-coding genes overexpressed in 21 cancer types. (**B**) Protein-coding genes underexpressed in 21 cancer types. Groups of cancer types discussed in the text are marked by different colors. BLCA, bladder urothelial carcinoma; BRCA, breast invasive carcinoma; COAD, colon adenocarcinoma; ESCA, esophageal carcinoma; GBM, glioblastoma multiforme; HNSC, head and neck squamous cell carcinoma; KIRC, kidney renal clear cell carcinoma; KIRP, kidney renal papillary cell carcinoma; LAML, acute myeloid leukemia; LIHC, liver hepatocellular carcinoma; LUAD, lung adenocarcinoma; LUSC, lung squamous cell carcinoma; OV, ovarian serous cystadenocarcinoma; PAAD, pancreatic adenocarcinoma; PRAD, prostate adenocarcinoma; READ, rectum adenocarcinoma; SKCM, skin cutaneous melanoma; STAD, stomach adenocarcinoma; TGCT, testicular germ cell tumors; THCA, thyroid carcinoma; UCEC, uterine corpus endometrial carcinoma.

To evaluate differences between full human and pan-cancer structuromes, we calculate over and underrepresentation of cancer-related (over and under expressed in 21 cancer types) protein domains in ECOD and ECOD_AF A-groups (Fig. 4A, B). In spite of the shared proteins (see “Materials and methods”), there were significant differences between the over and underexpressed protein sets. Heatmap analysis of the overexpressed protein-coding genes set revealed 4 major groups of cancer types (Fig. 4A). The first group is dominated by significant overrepresentation of protein domains from the “a + b duplicates or obligate multimers” ECOD A-group (BLCA, LUSC, BRCA, COAD, READ, OV, STAD, UCEC). The second group is dominated by significant overrepresentation of domains from the “beta sandwiches” A-group (LUAD, SKCM, KIRC, ESCA, LAML, TGCT, GBM, PAAD). The third group clusters KIRP, HNSC, LIHC and THCA. The final two cancer types did not show any significant overrepresentation for this protein set, whereas the first two are dominated by overrepresentation of domains from the “extended segments”, “mixed a + b and a/b”, and “beta sandwiches”. Finally, PRAD stands alone with significant overrepresentation by domains from the “a + b three layers” A-group. Heatmap analysis of underexpressed set revealed three major groups of cancer types (Fig. 4B). The first group is dominated by significant over representation of beta sandwiches, beta meanders, and extended segments. Conversely, there was significant under representation of protein domains from the “alpha arrays” A-group (GBM, COAD, READ, LUAD, UCEC, LUSC). The second group brings together cancer types with overrepresentation of protein domains from beta sandwiches, beta barrels and alpha duplicates A-groups (HNSC, ESCA, LAML, SKCM, TGCT, THCA, PRAD, OV, BRCA, BLCA). Finally, the third group is dominated by overrepresentation of a/b barrels, alpha complex topologies, and underrepresentation of protein domains from alpha bundles and alpha arrays A-groups.

**Figure 5:**
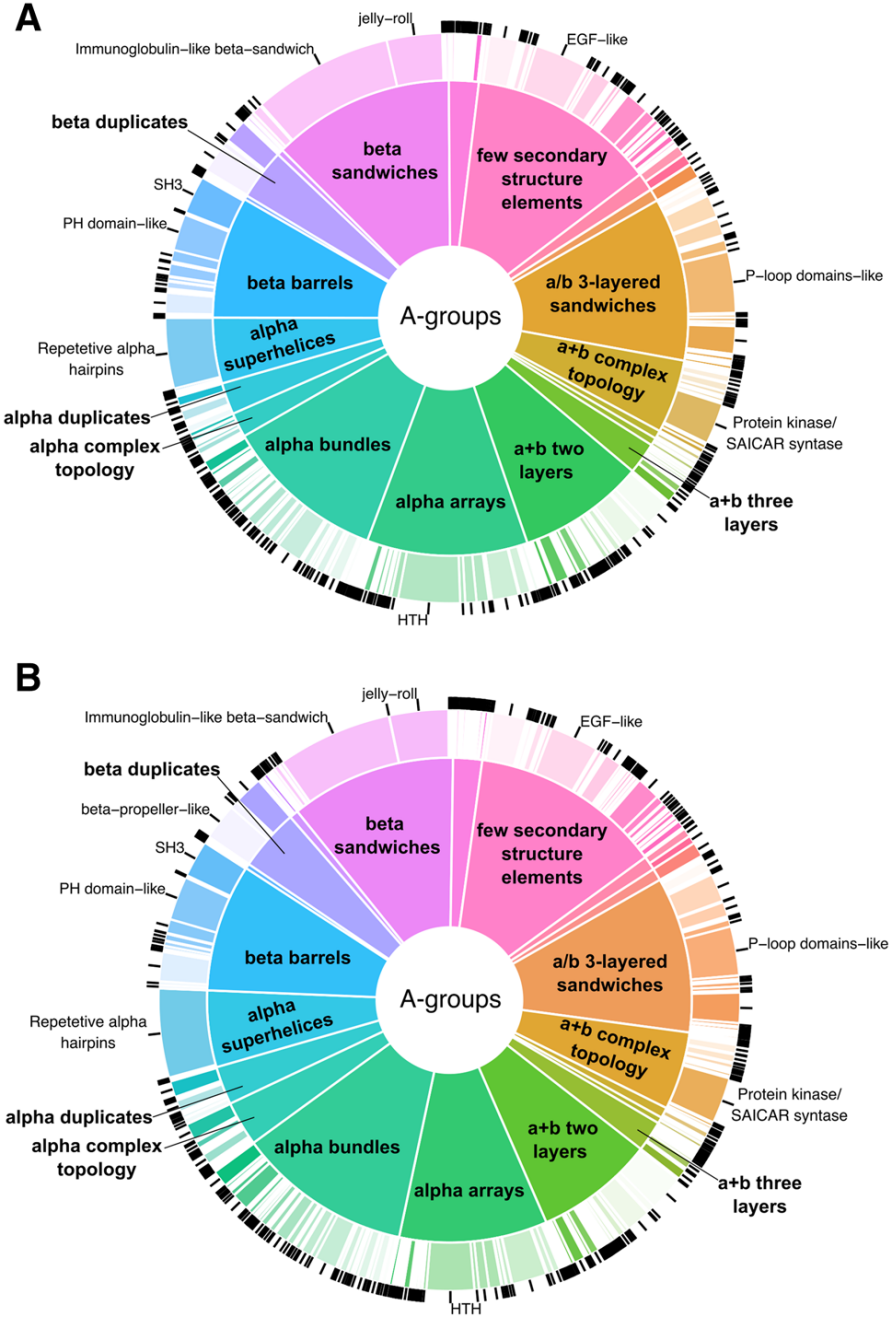
Distribution of ECOD A- and X-groups for all cancer types. (**A**) Overexpressed protein-coding genes set. (**B**) Underexpressed protein-coding genes set. Inner pie chart corresponds to A-groups, outer—to X-groups. The largest groups are labeled.

**Figure 6:**
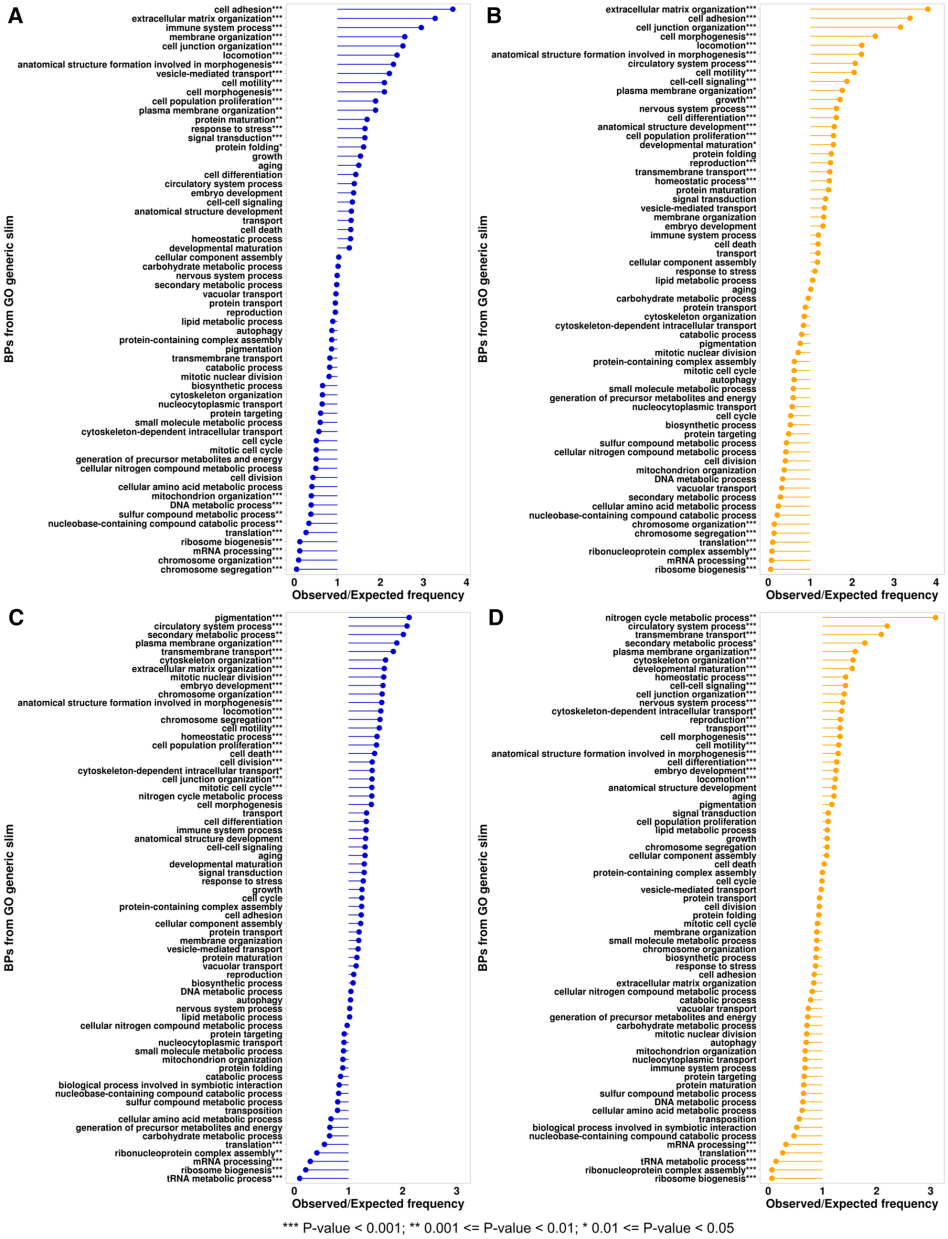
The ratio of observed and expected frequencies of Biological Processes (BPs) from GO generic subset defines over (ratio > 1) and under (ratio < 1) represented process. (**A**) Proteins containing domains from beta sandwiches A-groups in overexpressed set. (**B**) Proteins containing domains from beta sandwiches A-groups in underexpressed set. (**C**) Proteins containing domains from five exclusively alpha A-groups in overexpressed set. (**D**) Proteins containing domains from five exclusively alpha A-groups in underexpressed set.

**Figure 7:**
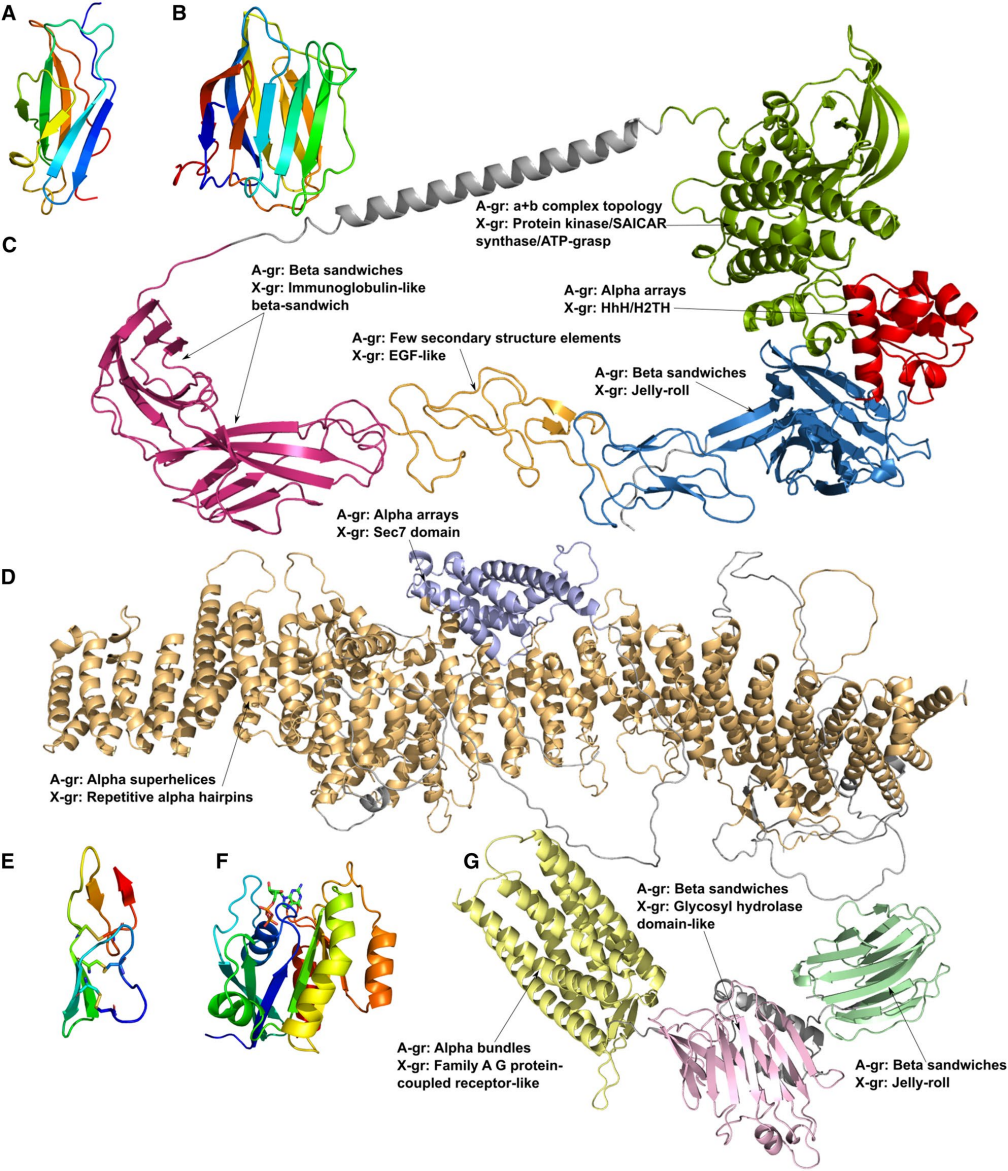
Representative domain structures from the largest A-groups. (**A**) Immunoglobulin-like beta sandwich domain of the cell surface interleukin-10 (PDB: 1Y6N). (**B**) Jelly-roll domain of the galectin GAL1 (PBD: 6M5Y). (**C**) AlphaFold model of ephrin type-A receptor 1 (P21709). (**D**) AlphaFold model of brefeldin A-inhibited guanine nucleotide-exchange protein 3 (Q5TH69). (**E**) EGF-like domain of Mucin-4 (AlphaFold: Q99102). Disulfide bonds are shown as sticks. (**F**) P-loop domains of Ras-related protein Rab-11A (PDB: 1OIV). Domain structures are colored in rainbow. (**G**) AlphaFold model of adhesion G-protein coupled receptor G1 (Q9Y653).

**Figure 8:**
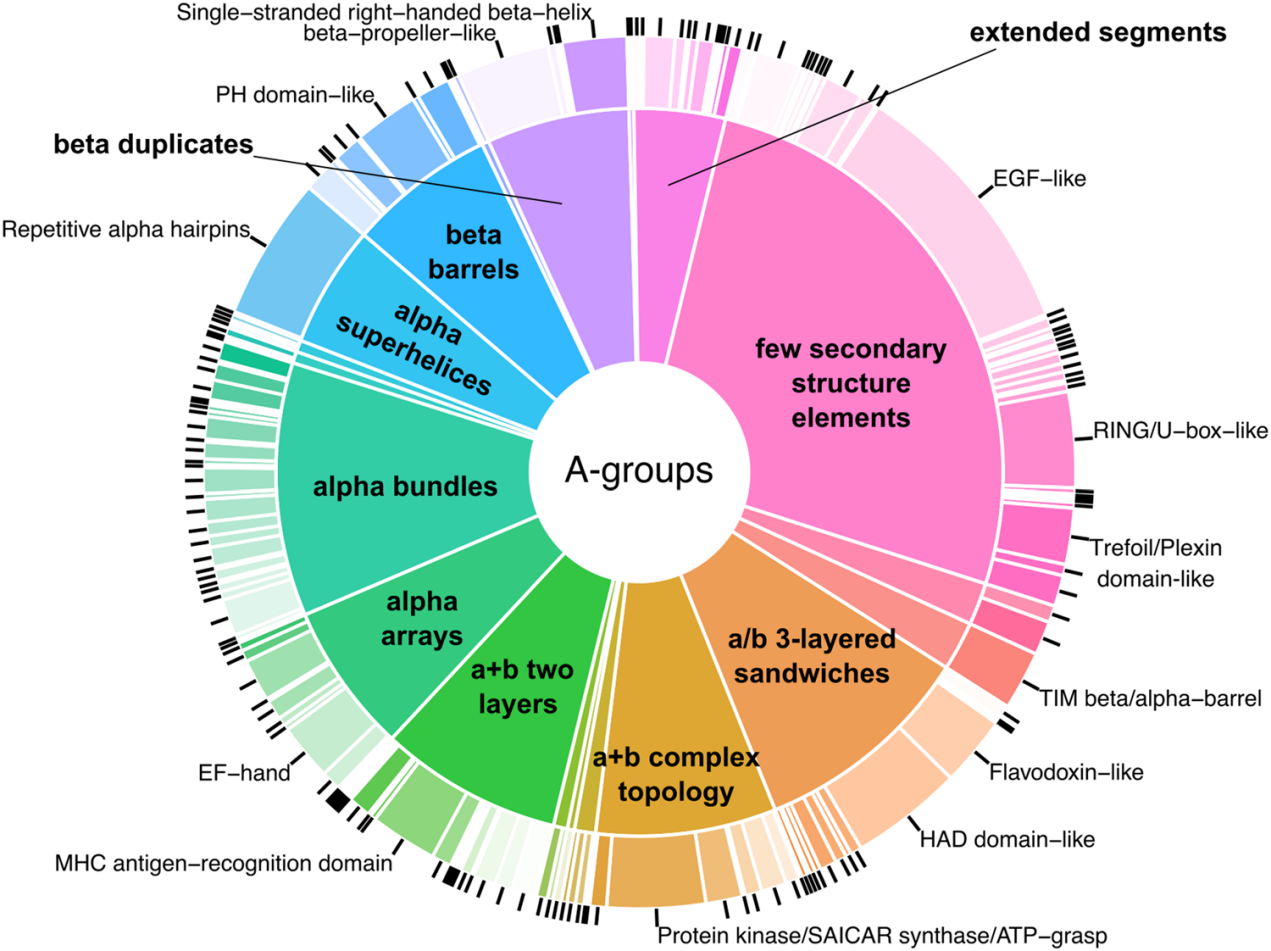
Distribution of domain contexts of beta sandwiches ECOD A-group in overexpressed dataset.

In spite of major differences described above between the structuromes of 21 cancer types there are couple common features. The first feature is that the beta sandwiches are the most overrepresented ECOD architecture in both (over and underexpressed) protein sets (Fig. 4). The majority of the domains from this A-group belongs to Immunoglobulin-like beta sandwich X-group (Fig. 5). Immunoglobulins or antibodies are the key elements of inflammation. Inflammatory cells are the main components of the tumor microenvironment, which can be a crucial element of tumor progression^21,22^. Therefore, it is not surprising that the “immune cell” process is in the top three processes significantly overrepresented in the overexpressed set of the beta sandwiches A-group, but not the underexpressed group (Fig. 6A, B). For this analysis we used Gene Ontology “biological processes” (GO_BP) terms for each protein in over and underexpressed sets. GO_BP terms were mapped to GO terms from a generic slim subset that includes 69 top level biological processes. Over and underrepresentation of proteins expressed in different cancer types in BPs was calculated as ratio of observed and expected frequencies (see “Materials and methods”). Cell surface interleukin-10 (IL10) receptor subunit alpha (UniProt ID: Q13651), which is over expressed in many cancer types, is another important element of inflammatory processes that includes domains from the Immunoglobulin-like beta sandwich X-group (Fig. 7A)^23,24^. The jelly-roll is the second largest X-group in the beta sandwiches architecture (Fig. 5). Galectins are group of glycan-binding proteins that share the β-sandwich fold from the jelly-roll X-group (Fig. 7B)^25^. These proteins help reprogram the fate and function of various cell types and due to their multifunctional role in tissue fibrosis and cancer, they are considered potential therapeutic targets^26^. Three galectins are of special therapeutic relevance: GAL1 (P09382), GAL3 (P17931), and GAL9 (O00182), which are in our overexpressed proteins set. The proteins mentioned earlier, which contain beta sandwiches domains discussed above, exclusively consist of domains from a single ECOD A-group. Overall, 62% of proteins include domains from a single A-group and 38% from multiple A-groups in overexpressed dataset (61% and 39% in underexpressed dataset respectively) (SI Fig. 4A, B). Beta sandwiches domains make up 14.4% of all proteins that possess a single A-group (SI Fig. 4A). However, the beta sandwiches architecture group can also be observed within the context of a wide range of other A-groups in multidomain proteins (Fig. 8). The top three A-groups that can be observed within the context of beta sandwiches are few secondary structure elements, alpha bundles and a/b three-layered sandwiches (Fig. 8). For example, ephrin type-A receptor 1 (gene name: EPHA1, UniProt ID: P21709) is a receptor tyrosine kinase which binds membrane-bound ephrin-A family ligands. This protein plays a role in apoptosis, regulates cell proliferation and tumor angiogenesis^27^. It is overexpressed in several cancer types including hepatocellular carcinoma (HCC). So far, structures of only two regions of this protein have been determined using experimental approaches^28^. The AlphaFold model of EPHA1 (UniProt ID: P21709) contains six domains: three beta sandwiches (two Immunoglobulin-like and one jelly roll), one from few secondary structure elements A-group (EGF-like), one from alpha arrays (HhH/H2TH) and one form a + b complex topology (Protein kinase/SAICAR synthase/ATP-grasp) (Fig. 7C).

The second common feature is related to exclusively alpha architectures. There are five A-groups in ECOD that include exclusively alpha domains: alpha superhelices, alpha duplicate or obligate multimers, alpha complex topology, alpha bundles, and alpha arrays. Domains from these A-groups are mostly underrepresented (in many cases significantly, Fig. 4) in all cancer types in the overexpressed dataset. Exclusively alpha domains make up 32.8% of all proteins that possess a single A-group in overexpressed set and 36.8% in underexpressed set (SI Fig. 4). For example, transforming acidic coiled-coil-containing protein 3 (TACC3, UniProt ID: Q9Y6A5) represents the alpha bundles A-group with long disordered extensions at the N- and C-terminal ends. TACC3 plays a role in the microtubule-dependent coupling of the nucleus and the centrosome and is important in the development of multiple myeloma, breast and gastric cancer^29^. Since exclusively alpha helical domains include five ECOD A-groups, there are proteins that exclusively possess these architectural features within the context of multidomain proteins (4.8% and 5.7% in over and underexpressed protein sets respectively) (SI Fig. 4). For example, brefeldin A-inhibited guanine nucleotide-exchange protein 3 (ARFGEF3, UniProt ID: Q5TH69) adopts two domains that are classified in alpha superhelices and alpha arrays A-groups (Fig. 7D). This protein plays a critical role in activation of the estrogen/ER signaling in breast cancer cells^30^. Alpha arrays stand out in the exclusively alpha A-groups (Fig. 4A). Domains from alpha arrays A-group show slight overrepresentation however it is significant only in one case (COAD). Alpha bundles and alpha arrays are the top two most populated A-groups in the human proteome (SI Fig. 3) and in over and underexpressed sets (Fig. 5A, B). We also calculated the over and underrepresentation of A-groups for all 21 cancer types in three different datasets: ECOD and ECOD_AF combined, only ECOD, and only ECOD_AF (SI Fig. 5). This analysis revealed that all five exclusively alpha A-groups are mostly underrepresented (in some cases statistically significantly, SI Fig. 5) in all three datasets. The alpha duplicates or obligate multimers A-group showed the highest ratio of observed/expected frequency, but its value is around 1.0 for all three datasets. Moreover, we calculated combined over and underrepresentation of domains from five exclusively alpha A-groups for each cancer type in three datasets mentioned above (SI Table 1). This analysis showed that overexpressed protein-coding genes with domains from five exclusively alpha A-groups are underrepresented in 20 cancer types and in 11 out of 20 underrepresentation is statistically significant (SI Table 1). Therefore, protein-coding genes overexpressed in 21 cancer types are mostly depleted in exclusively alpha-helical domains at the level of ECOD A-groups. In the underexpressed set 3 out of 5 exclusively alpha A-groups are still mostly underrepresented (alpha bundles, alpha arrays, alpha superhelices). However, the “alpha complex topology” and “alpha duplicate or obligate multimers” A-groups show significant overrepresentation in several cancer types (Fig. 4B). We also calculated combined over and underrepresentation of domains from five exclusively alpha A-groups for each cancer type in the underexpressed set (SI Table 2). For the underexpressed set three cancer types showed overrepresentation of domains from the five exclusively alpha A-groups (GBM, SKCM, TGCT) and one of them is statistically significant (GBM). The rest 18 cancer types show underrepresentation and 10 of them are statistically significant (SI Table 2). Therefore, the underexpressed set also showed that exclusively alpha A-groups were underrepresented in most cancer types, however to a lesser extent than in the overexpressed set.

Alpha helical domains are known to constitute the majority of transmembrane domains^31^. Although beta barrels can also be transmembrane domain, for example, in the outer membranes of bacteria, mitochondria, and chloroplasts, these cases are less common^32^. Proteins containing transmembrane domains are part of the surfaceome—a broader set of proteins that are linked to the cellular membrane^33^. To evaluate the functional distribution of proteins containing domains from the exclusively alpha A-groups, we mapped GO_BP ids retrieved from UniProt KB^34^ to the GO generic slim subset and calculated over and underrepresentation (Fig. 6C, D). We also retrieved annotations regarding transmembrane and intramembrane regions in each protein from UniProt and noted if any particular protein is included in surfaceome from the overexpressed set (SI Table 3). The overexpressed set is 29% (527 out of 1975) composed of proteins that contain trans and/or intramembrane regions and 24% (469 out of 1975) composed of proteins included in the surfaceome (SI Table 3). Not all proteins containing transmembrane domains are included in the surfaceome since genes encoding proteins in intracellular membranes are not considered (nuclear and mitochondrial)^33^. Membrane proteins play an important role in the function of any cell in the body by controlling communications between cells and the extracellular environment^35^. Due to the critical biological function of membrane proteins (especially those included into surfaceome), these proteins are a valuable resource for identifying targets for immune and targeted therapy^36,37^. Recently, powerful treatment approaches have been developed for multiple types of cancer that are based on targeting membrane proteins by chimeric antigen receptor T cells (CAR-Ts)^38^ or antibodies^39^. The functional distribution of proteins containing domains from exclusively alpha A-groups (Fig. 6C, D) revealed significant overrepresentation of these proteins in processes related to the membrane and extracellular matrix (plasma membrane organization, transmembrane transport, extracellular matrix organization), processes related to the cytoskeleton, as it contains fibrillar alpha proteins (cytoskeleton organization, cytoskeleton-dependent intracellular transport, cell motility, locomotion), homeostasis and cell death. Therefore, our analysis revealed an underrepresentation of proteins containing domains from five exclusively alpha ECOD A-groups in both the overexpressed and underexpressed sets, to a lesser extent in the latter. This observation suggests that on average expression level of proteins with exclusively alpha domains remains unaltered during the transition from normal to cancer cell. Homeostasis, apoptosis and transmembrane transport are highly interconnected processes in cellular biology. Alterations in the transmembrane gradients of various physiological ions can have a significant impact on programmed cell death, including apoptosis^40,41^. At the same time, one of the most important hallmarks of cancer is enabling replicative immortality^42^. Therefore, it is possible that cancer cells reduce representation of proteins that are related to such biological processes as homeostasis, apoptosis and transmembrane transport to promote their survival and growth. The reduction in the representation of proteins with exclusively alpha domains in cancer could suggest a disruption in these important cellular processes, contributing to cancer cell survival and proliferation. We believe that it is the main reason for underrepresentation of proteins with exclusively alpha domains in most of the cancer types.

Moreover, we studied subcellular location of all proteins from over and underexpressed sets using UniProt annotation. The analysis conducted revealed that the majority of the studied proteins are associated with membranes, followed by cytoplasm and nucleus as the second and third most prevalent locations, respectively (SI Fig. 6A, B). It should be noted that due to the existence of protein isoforms one protein could be assigned to several subcellular locations. For example, isoforms 1, 2, 6 and 7 of complement decay-accelerating factor (CD55, UniProt ID: P08174) are membrane-associated, however isoforms 3, 4, and 5 are secreted^43^. However, not only exclusively alpha-helical proteins are associated with the membrane. Proteins that contain beta sandwiches, including various receptors, exhibit a similar association with membranes. For example, interleukin-11 receptor subunit alpha (IL11RA, UniProt ID: Q14626)^44^, triggering receptor expressed on myeloid cells 1 (TREM1, UniProt ID: Q9NP99)^45^ and many others. Moreover, the membrane-associated subcellular location contains the largest number of beta sandwiches-containing proteins than any other category for both over and underexpressed protein datasets (SI Fig. 6A, B).

The other largest A-groups include “few secondary structure elements” and “a/b three-layered sandwiches” (Fig. 5). The EGF-like X-group is the most populated from the few secondary structure architecture in the over and underexpressed sets. Mucins belong to *O*-glycoproteins functional category and include EGF-like domains in their structural organization and are characterized by multiple disulfide bonds (Fig. 7E)^46^. Expression of these protein-coding genes is often altered in epithelial cancers^47^. Mucins are also important therapeutic targets due to their role in inflammation^48^. “P-loop domains-like” is the largest X-group in “a/b three-layered sandwiches” architecture (Fig. 5). P-loop domains adopt a Rossmann-like fold (Fig. 7F), which is one of the most prominent structural units in nature, and Rossmann-like proteins are known to be a key element of the majority of metabolic pathways^49,50^.

The combination of exclusively alpha helical and beta sandwiches A-groups within multidomain protein constitute a small fraction of 3.6% in overexpressed and 4.3% in underexpressed protein sets (SI Fig. 4). Adhesion G-protein coupled receptor G1 (ADGRG1, UniProt ID: Q9Y653) adopts two beta sandwiches domains and one domain from alpha bundles A-group (Fig. 7G). ADGRG1 plays a critical role in melanoma progression by inhibiting angiogenesis through a signaling pathway mediated by protein kinase C alpha type^51^.

The newly predicted AF protein structures and their classification into evolutionary units (domains) offer additional opportunities for cancer related research. One of the major applications is the identification of potential targets for therapeutic intervention and design of novel cancer treatments. Indeed, AlphaFold-Multimer has been demonstrated to achieve state-of-the-art performance in peptide-protein docking and peptide-protein interaction prediction^52^. Notably, it has been successfully employed as an integral component of an AI-powered drug discovery approach to identify de novo molecules capable of inhibiting cyclin-dependent kinase 20 in hepatocellular carcinoma^53^. This showcases the potential of AF approach in accelerating the discovery of effective therapeutics for cancer treatment. Identification of domains within newly predicted AF structures can aid in the discovery of potential drug targets by detecting distant homology. The ECOD database serves as a valuable tool for this task, as it allows for the classification and analysis of protein domains based on evolutionary relationships. Another valuable application of AF is pocket prediction, which has been demonstrated to be highly accurate for confident models^54^. The ability to predict pockets in proteins accurately can aid in understanding protein–ligand interactions and facilitate drug discovery efforts by identifying potential binding sites for small molecule drugs.

## Conclusions

Analysis of the structural space of cancer-related proteins (both over and underexpressed) revealed significant differences between 21 major cancer types. We have shown that AlphaFold models significantly expanded the structurome of protein-coding genes differentially expressed in 21 cancer types and should be considered in structured-based analyses of cancer proteins. We evaluated the pan-cancer structurome at the two top levels of ECOD classification: A-groups (architecture) and X-groups (possible homology). At the architecture level the majority of cancer types in both protein sets showed significant overrepresentation of the beta sandwiches architecture. Proteins that contain domains adopting beta sandwich folds include the immunoglobulins, interleukins and galectins, which are crucial elements of inflammatory processes and they play an important role in oncogenesis. Moreover, we showed that domains from the five exclusively alpha A-groups are significantly underrepresented in the majority of cancer types. Alpha-helical domains compose the majority of transmembrane domains and are the part of the surfaceome. These proteins are important therapeutic target for cancer treatment. Moreover, proteins with exclusively alpha domains are important elements of homeostasis, apoptosis and transmembrane transport, which are closely related processes. Changes in the transmembrane gradients of various physiological ions can have an impact on the regulation of programmed cell death. In order to attain a crucial hallmark of cancer known as replicative immortality, cancer cells reduce representation of proteins that are related to biological processes mentioned above. This reduction leads to underrepresentation of proteins with exclusively alpha domains among other cancer-related proteins in 21 cancer types.

## Materials and methods

### Data collection

Sets of significantly over- and underexpressed genes compared to the normal samples for 21 major cancer types were retrieved from Gene Expression Profiling Interactive Analysis web server (GEPIA2)^55^ using following cutoffs: Log2 Fold Change > 2, adjusted P-value < 0.005. The following cancer types were considered in current analysis: BLCA, bladder urothelial carcinoma; BRCA, breast invasive carcinoma; COAD, colon adenocarcinoma; ESCA, esophageal carcinoma; GBM, glioblastoma multiforme; HNSC, head and neck squamous cell carcinoma; KIRC, kidney renal clear cell carcinoma; KIRP, kidney renal papillary cell carcinoma; LAML, acute myeloid leukemia; LIHC, liver hepatocellular carcinoma; LUAD, lung adenocarcinoma; LUSC, lung squamous cell carcinoma; OV, ovarian serous cystadenocarcinoma; PAAD, pancreatic adenocarcinoma; PRAD, prostate adenocarcinoma; READ, rectum adenocarcinoma, SKCM, skin cutaneous melanoma; STAD, stomach adenocarcinoma; TGCT, testicular germ cell tumors; THCA, thyroid carcinoma; UCEC, uterine corpus endometrial carcinoma. Non-protein coding genes were filtered out using UniProt KB^34^. Overall non-redundant sets of over and underexpressed proteins for all cancer types include 5341 and 7320 proteins correspondingly (total over- and underexpressed proteins constitute a non-redundant list of 10,277 proteins). 2384 proteins belong to both sets (over and underexpressed), since the same proteins might be overexpressed in one cancer type and underexpressed in another type.

### Functional distribution of the proteins

For the functional analysis of the proteins over and underexpressed in different cancer types we applied the approach that we recently used^56^. Gene Ontology “biological processes” (GO_BP)^57^ information was retrieved for each protein in the over and underexpressed sets from UniProt KB^34^. Of 10,277 proteins in these sets, only 1381 have no GO_BP assignment. We used the DeepFRI approach^58^ to predict missing GO_BP assignments for these 1381 unassigned proteins. Most (1302) have no known 3D structure, so we used AlphaFold models (AlphaFold DB version 4, 2022-11-01)^7^ as input for the DeepFRI predictor. A DeepFRI score larger than 0.5 was considered significant. Overall, 616 out of 1381 (45%) proteins were assigned to GO_BP based on our DeepFRI prediction. Proteins with a predicted GO_BP assignment (616 proteins) were merged with known GO_BP assignments (8886 proteins) for further analysis. The predicted subset comprises 6.5% of the whole set (616 out of 9512 proteins) used for the functional distribution analysis. GO_BP terms were mapped to GO terms from a generic slim subset. The GO generic slim subset includes 69 top level biological processes (BPs). One protein can be involved in several BPs. Over and underrepresentation of proteins expressed in different cancer types in BPs was calculated as ratio of observed and expected frequencies. The observed frequency for each BP was calculated as a ratio of the number of the proteins assigned to this BP over the sum of all proteins assigned to any BP. The expected frequency for each BP was calculated as ratio of total number of proteins assigned to this BP in human proteome over the total number of proteins assigned to any BP in human proteome. The significance of the representation was checked using the chi-squared test. We considered three levels of significance: P-value < 0.001 (***), 0.001 ≤ P-value < 0.01 (**), 0.01 ≤ P-value < 0.05 (*). Statistical analysis was conducted using the R package, v4.2.1^59^.

### Protein domains data

Information about protein domains and their hierarchical classification was obtained from the Evolutionary Classification of Protein Domains (ECOD)^1,2^. ECOD is a protein classification of homologous domains with a five-level hierarchy: architecture (A), possible homology (X), homology (H), topology (T), and family (F). For each protein (UniProt ID) we collected all known PDB structures and retrieved their domain organization from ECOD. For proteins without known 3D structures domain data were retrieved from of the provisional ECOD human classification^10^ (ECOD_AF) that includes only human protein structures predicted by AlphaFold and distributed by UniProt. AlphaFold models have been classified to the T-group level (i.e., not into sequence families). The two ECOD domain classifications were merged. This merged set includes classification to the T-group level. The ECOD domain classification includes all experimentally identified protein structures (PDBs). Consequently, for some proteins (UniProt IDs) there are several PDBs for the same protein region, which results in redundant domains. In the merged set (ECOD and ECOD_AF), we eliminated redundancy in domains to ensure that each protein (UniProt ID) has no more than one domain representing the same protein region. Over and underrepresentation of proteins expressed in different cancer types, which domains belong to particular ECOD A-gr/X-gr, were calculated as ratio of observed and expected frequencies. The observed frequency for each A-gr/X-gr was calculated as a ratio of the number of the proteins assigned to this A-gr/X-gr in particular type of cancer over the sum of all proteins assigned to any A-gr/X-gr in this cancer type. The expected frequency for each A-gr/X-gr was calculated as ratio of total number of proteins assigned to this A-gr/X-gr in all human proteins classified in ECOD over the total number of proteins assigned to any A-gr/X-gr in all human proteins classified in ECOD. Significance of overrepresentation was checked using chi-square test. We considered three levels of significance: P-value < 0.001 (***), 0.001 ≤ P-value < 0.01 (**), 0.01 ≤ P-value < 0.05 (*).

## Supplementary Information

Supplementary Information 1.

Supplementary Table 3.

## Data Availability

All generated data are included in this manuscript and supplementary materials.
