# Supplementary material for: Pan-cancer structurome reveals overrepresentation of beta sandwiches and underrepresentation of alpha helical domains
Source: Sci Rep. 2023 Jul 25;13:11988. doi: 10.1038/s41598-023-39273-5 (PMC10368619; doi:10.1038/s41598-023-39273-5)
Supplement: Supplementary file 1 — Supplementary Information 1. [file 41598_2023_39273_MOESM1_ESM.pdf]

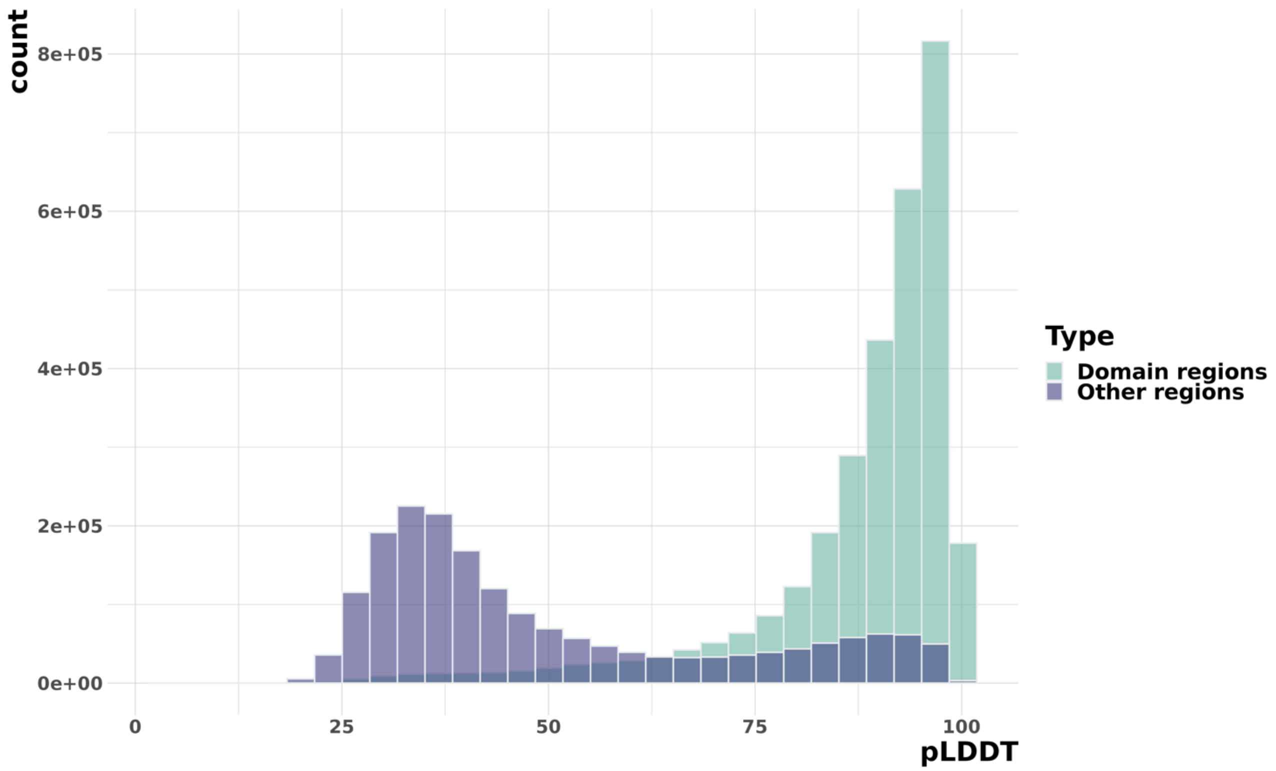


**SI Figure 1. Distributions of pLDDT score per residue for AlphaFold models of protein-coding genes over and underexpressed in 21 cancer types.**

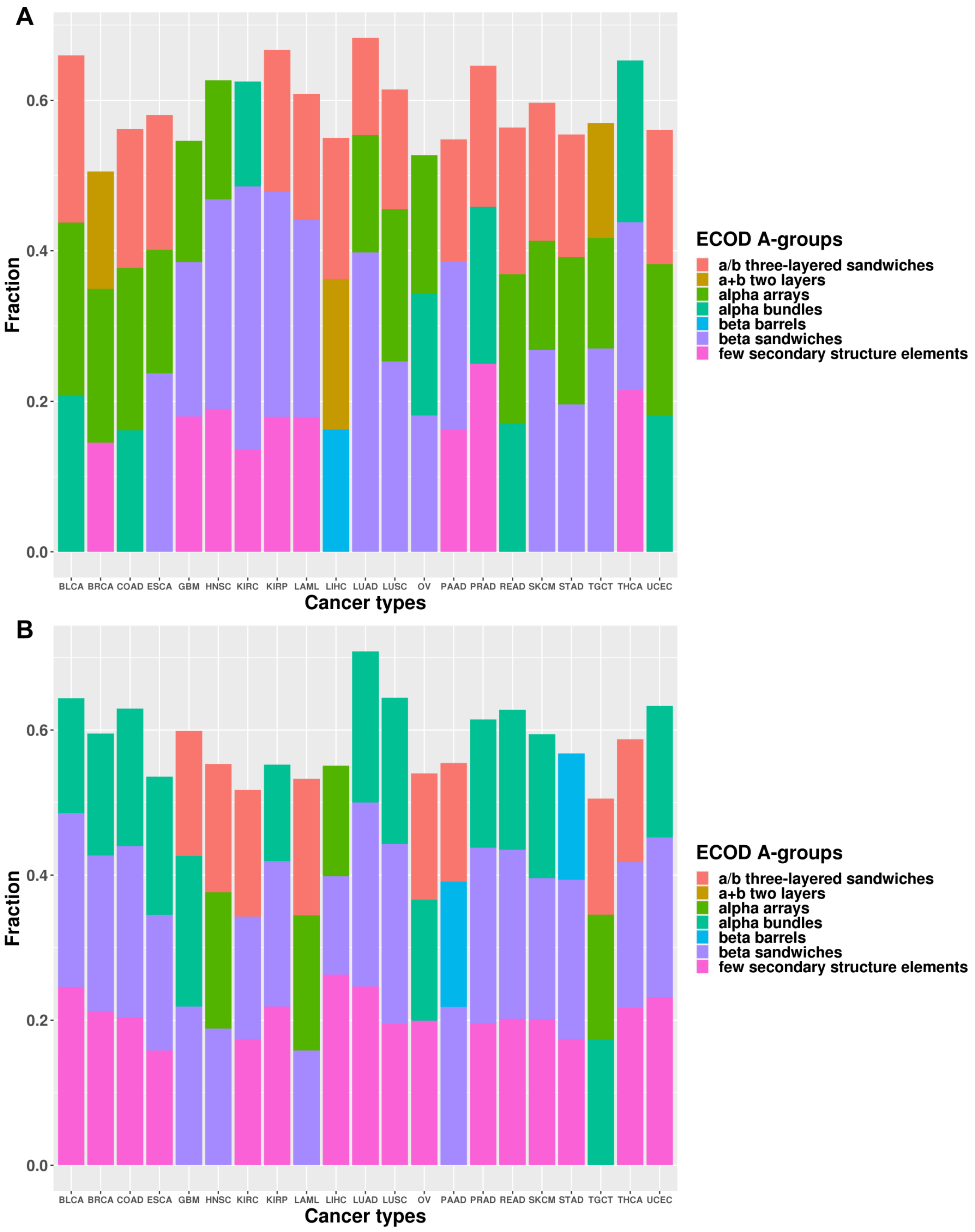


**SI Figure 2. Distribution of cancer-related proteins in top three ECOD A-groups for each cancer type.** **(A)** Protein-coding genes overexpressed in 21 cancer types. **(B)** Protein-coding genes underexpressed in 21 cancer types. Abbreviations: BLCA - Bladder urothelial carcinoma, BRCA - Breast invasive carcinoma,

COAD - Colon adenocarcinoma, ESCA - Esophageal carcinoma, GBM - Glioblastoma multiforme, HNSC - Head and Neck squamous cell carcinoma, KIRC - Kidney renal clear cell carcinoma, KIRP - Kidney renal papillary cell carcinoma, LAML - Acute Myeloid Leukemia, LIHC - Liver hepatocellular carcinoma, LUAD - Lung adenocarcinoma, LUSC - Lung squamous cell carcinoma, OV - Ovarian serous cystadenocarcinoma, PAAD - Pancreatic adenocarcinoma, PRAD - Prostate adenocarcinoma, READ - Rectum adenocarcinoma, SKCM - Skin Cutaneous Melanoma, STAD - Stomach adenocarcinoma, TGCT - Testicular Germ Cell Tumors, THCA - Thyroid carcinoma, UCEC - Uterine Corpus Endometrial Carcinoma.

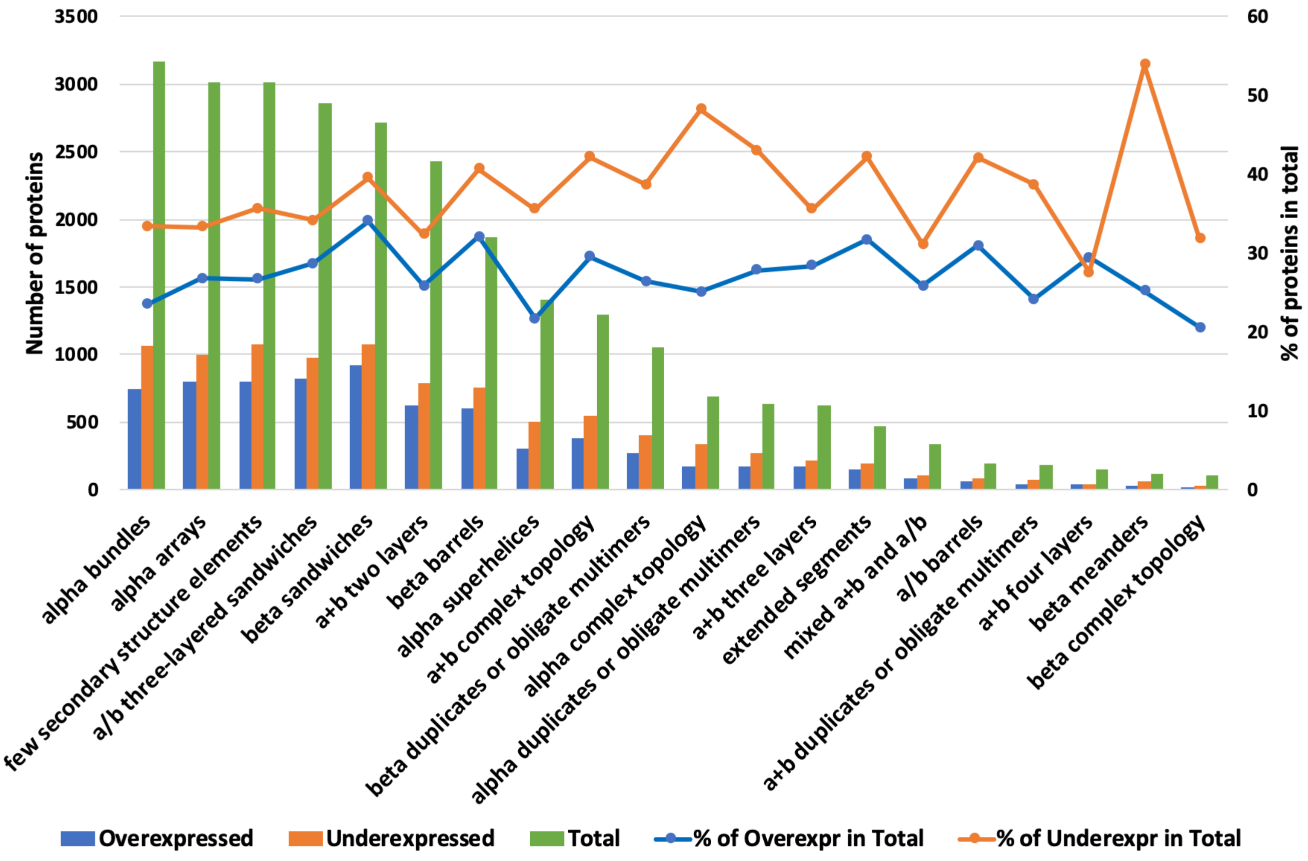


**SI Figure 3. ECOD A-groups statistics for over and underexpressed protein-coding genes in 21 cancer types.** Total (green color) represents the overall number of proteins which domains are assigned to particular A-group in ECOD and ECOD_AF.

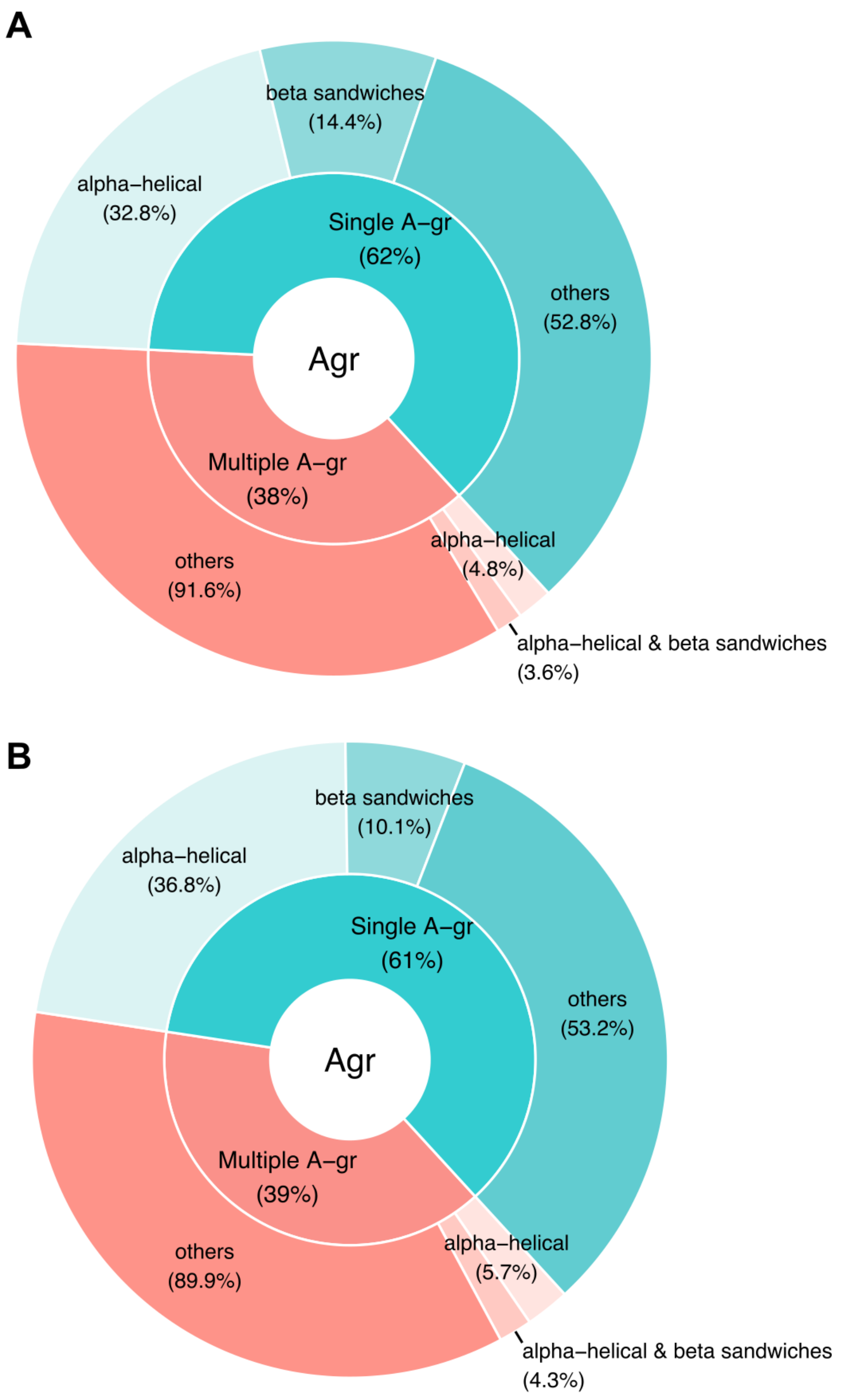


**SI Figure 4. Distribution of cancer-related proteins with single and multiple ECOD A-groups. (A)** Protein-coding genes overexpressed in 21 cancer types. **(B)** Protein-coding genes underexpressed in 21 cancer types. Alpha-helical category includes only five exclusively alpha A-groups: alpha superhelices, alpha duplicate or obligate multimers, alpha complex topology, alpha bundles, and alpha arrays. Beta sandwiches category includes all proteins that contain beta sandwiches and might contain domains form other ECOD A-groups.

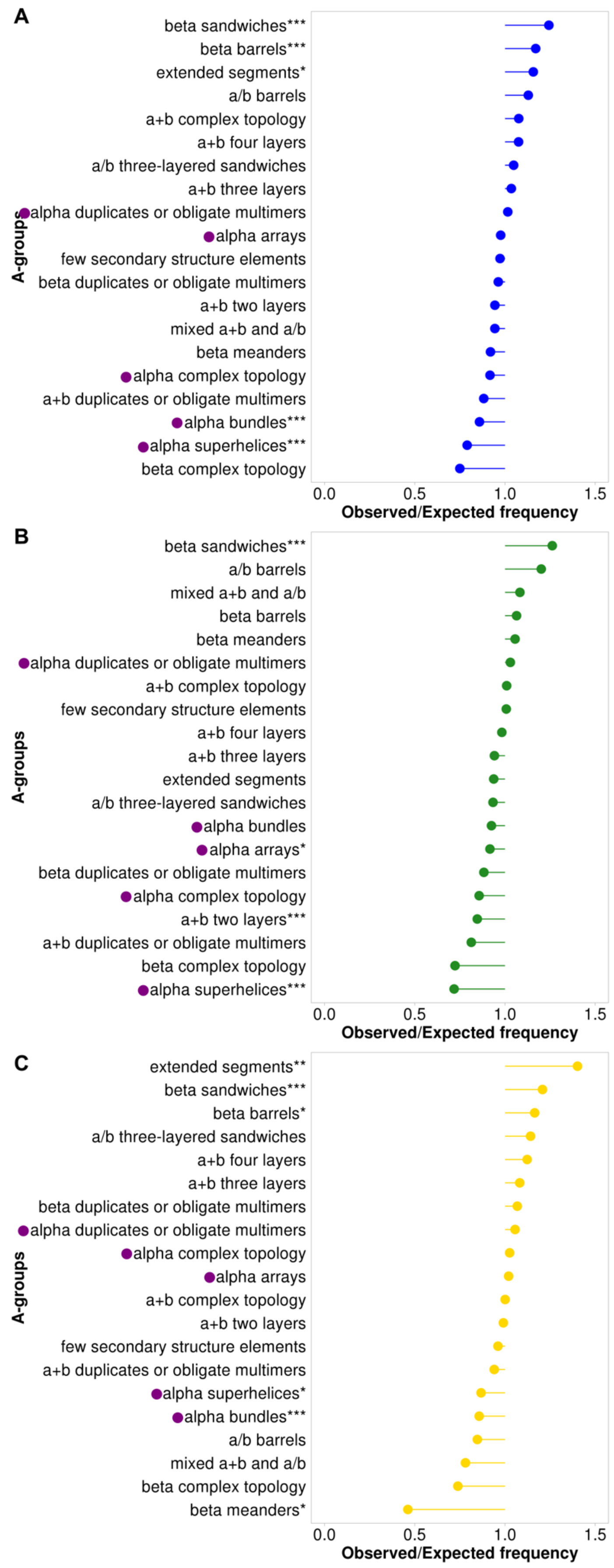


*** P-value < 0.001; ** 0.001 <= P-value < 0.01; * 0.01 <= P-value < 0.05

**SI Figure 5. Over and under representation of domains from ECOD A-groups in over expressed proteins set. (A)** Domains data from ECOD and ECOD_AF. **(B)** Domains data from ECOD only (without AlphaFold2 models). **(C)** Domains data from ECOD_AF only (only AlphaFold2 models). A-groups that include exclusively alpha domains are marked by purple spheres.

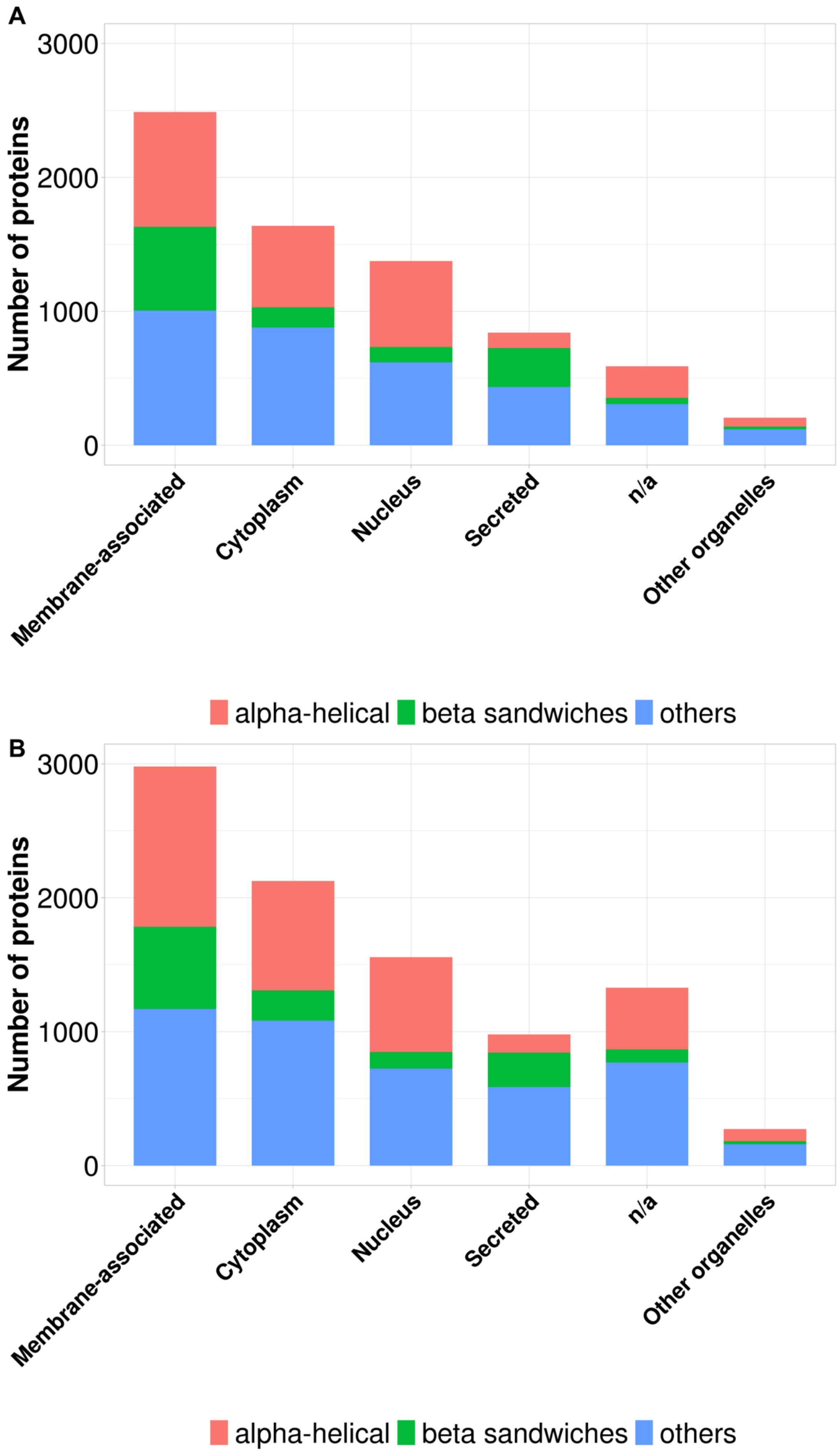
A
Number of proteins
3000
2000
1000
0
Membrane-associated
Cytoplasm
Nucleus
Secreted
n/a
Other organelles
alpha-helical
beta sandwiches
others
B
Number of proteins
3000
2000
1000
0
Membrane-associated
Cytoplasm
Nucleus
Secreted
n/a
Other organelles
alpha-helical
beta sandwiches
others

**SI Figure 6. Subcellular location of cancer-related proteins based on UniProt annotation. (A)** Protein-coding genes overexpressed in 21 cancer types. **(B)** Protein-coding genes underexpressed in 21 cancer types. Alpha-helical category includes only five exclusively alpha A-groups: alpha superhelices, alpha duplicate or obligate multimers, alpha complex topology, alpha bundles, and alpha arrays. Beta sandwiches category includes all proteins that contain beta sandwiches and might contain domains form other ECOD A-groups.

| | ECOD+ECOD_AF | | ECOD only | | ECOD_AF only | |
|---|---|---|---|---|---|---|
| Cancer types | Obs/Exp | Chi-square P-value | Obs/Exp | Chi-square P-value | Obs/Exp | Chi-square P-value |
| BLCA | 1.092801 | 0.298215 | 1.265052 | 0.019517 | 0.879673 | 0.455532 |
| BRCA | 0.903987 | 0.220682 | 0.909554 | 0.368815 | 1.028075 | 0.839044 |
| COAD | 0.976618 | 0.585335 | 1.015586 | 0.799243 | 0.981766 | 0.768184 |
| ESCA | 0.865412 | 0.036698 | 0.806161 | 0.03858 | 0.967341 | 0.72015 |
| GBM | 0.829254 | 6.56E-08 | 0.859308 | 0.00113 | 0.848949 | 0.001808 |
| HNSC | 0.709458 | 6.43E-04 | 0.781673 | 0.078559 | 0.663081 | 0.005051 |
| KIRC | 0.731385 | 2.49E-05 | 0.695233 | 5.14E-04 | 0.837691 | 0.095877 |
| KIRP | 0.681705 | 0.001311 | 0.542165 | 0.001016 | 0.901692 | 0.50314 |
| LAML | 0.936049 | 0.016266 | 0.977834 | 0.588629 | 0.919493 | 0.022501 |
| LIHC | 0.727536 | 0.023072 | 0.847133 | 0.326897 | 0.588605 | 0.045512 |
| LUAD | 0.625837 | 1.82E-06 | 0.767403 | 0.043424 | 0.504518 | 6.18E-06 |
| LUSC | 0.846682 | 0.00327 | 0.926393 | 0.32965 | 0.798359 | 0.006501 |
| OV | 0.934554 | 0.110161 | 0.908945 | 0.119156 | 1.012628 | 0.831439 |
| PAAD | 0.810969 | 0 | 0.835603 | 5.19E-08 | 0.83212 | 5.74E-07 |
| PRAD | 0.898192 | 0.511153 | 0.658881 | 0.216053 | 1.009037 | 0.960816 |
| READ | 0.952574 | 0.234399 | 0.958253 | 0.462934 | 0.995401 | 0.936536 |
| SKCM | 0.747528 | 1.11E-07 | 0.762114 | 5.69E-04 | 0.764841 | 4.86E-04 |
| STAD | 0.911156 | 0.064847 | 0.967212 | 0.615142 | 0.90584 | 0.211564 |
| TGCT | 0.638471 | 0 | 0.604621 | 2.29E-12 | 0.73533 | 2.98E-05 |
| THCA | 0.908585 | 0.347879 | 0.790658 | 0.135799 | 1.086655 | 0.534631 |
| UCEC | 0.984841 | 0.746486 | 1.00985 | 0.881164 | 1.013911 | 0.841099 |

**SI Table 1. Over and under representation of domains from five alpha exclusive A-groups in over expressed proteins set.** Sections of the table: ECOD+ECOD_AF - domains data from ECOD and ECOD_AF, ECOD only - domains data from ECOD only (without AlphaFold2 models), ECOD_AF only - domains data from ECOD_AF only (only AlphaFold2 models). Significant P-values < 0.01 are shown in red, over representation (Obs/Exp frequency > 1) – in blue, under representation (Obs/Exp frequency < 1) - in green.

| | ECOD+ECOD_AF | |
|---|---|---|
| Cancer types | Obs/Exp | Chi-square P-value |
| BLCA | 0.79939116 | 3.37E-05 |
| BRCA | 0.88355511 | 0.02738049 |
| COAD | 0.889472 | 0.00923538 |
| ESCA | 0.84140338 | 0.00875973 |
| GBM | 1.10050604 | 0.01077408 |
| HNSC | 0.86226462 | 0.0931789 |
| KIRC | 0.89305979 | 0.06006473 |
| KIRP | 0.89819232 | 0.06053141 |
| LAML | 0.92816429 | 0.03624557 |
| LIHC | 0.87687928 | 0.21188422 |
| LUAD | 0.91381305 | 0.05553646 |
| LUSC | 0.90475718 | 0.00623403 |
| OV | 0.96836359 | 0.33148855 |
| PAAD | 0.66629539 | 0.00109013 |
| PRAD | 0.94398251 | 0.51734925 |
| READ | 0.92477559 | 0.06485899 |
| SKCM | 1.00622998 | 0.84034005 |
| STAD | 0.79272715 | 0.0158932 |
| TGCT | 1.02076096 | 0.31684405 |
| THCA | 0.87406178 | 0.01746186 |
| UCEC | 0.89425421 | 4.46E-04 |

**SI Table 2. Over and under representation of domains from five alpha exclusive A-groups in under expressed proteins set.** ECOD+ECOD_AF - domains data from ECOD and ECOD_AF. Significant P-values < 0.01 are shown in red, over representation (Obs/Exp frequency > 1) – in blue, under representation (Obs/Exp frequency < 1) - in green
